# Supplementary material for: Cell surface remodeling caused by the loss of the flippase subunit TMEM30A in immune cells
Source: J Cell Sci. 2026 Aug 14;139(21):jcs264813. doi: 10.1242/jcs.264813 (PMC13535326; doi:10.1242/jcs.264813)
Supplement: Supplementary file 1 [file joces-139-264813-s1.pdf]

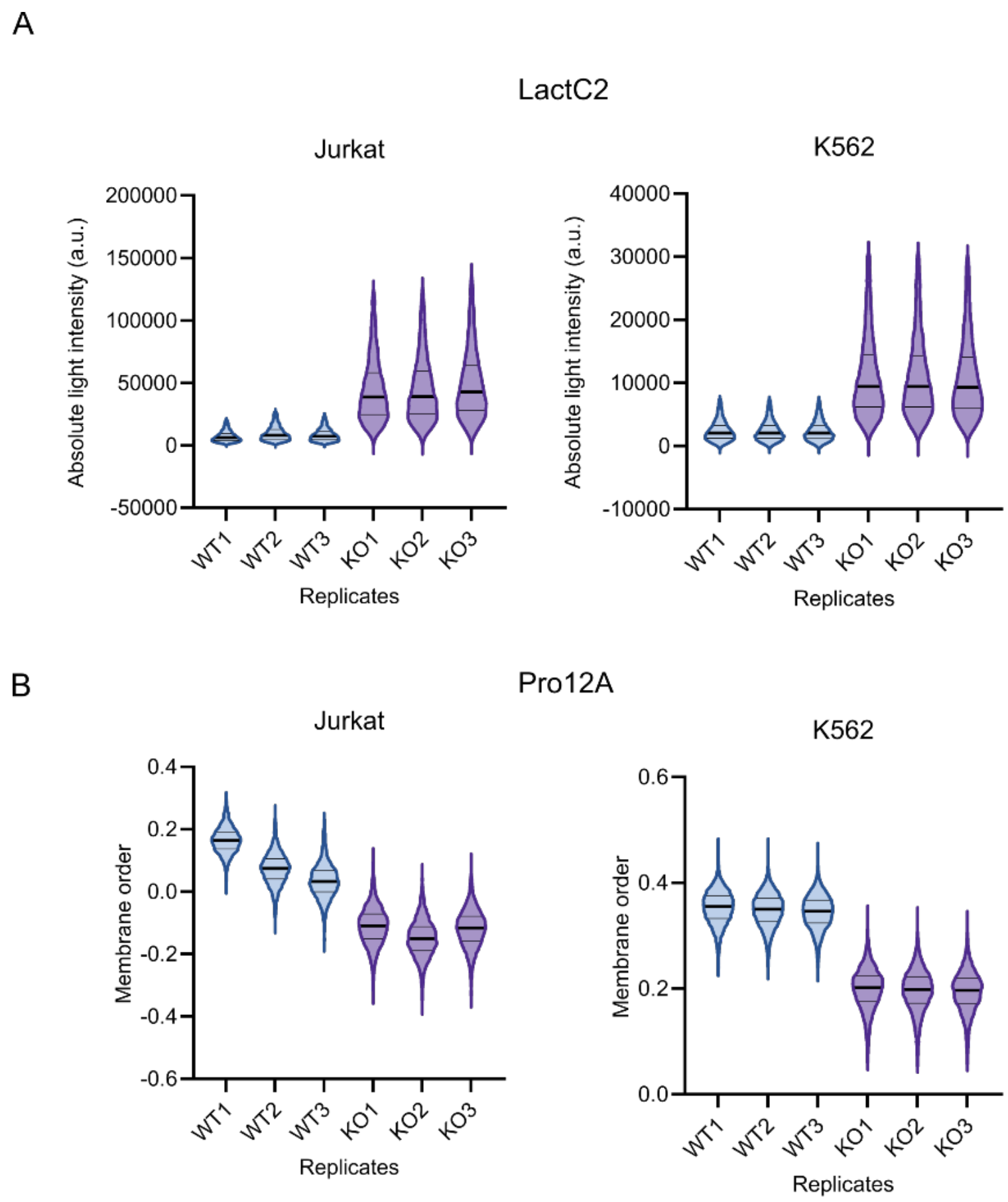

Fig. S1. A) Absolute light intensity measurements of LactC2 and B) membrane order of Pro12A for all replicates with spectral flow cytometry.

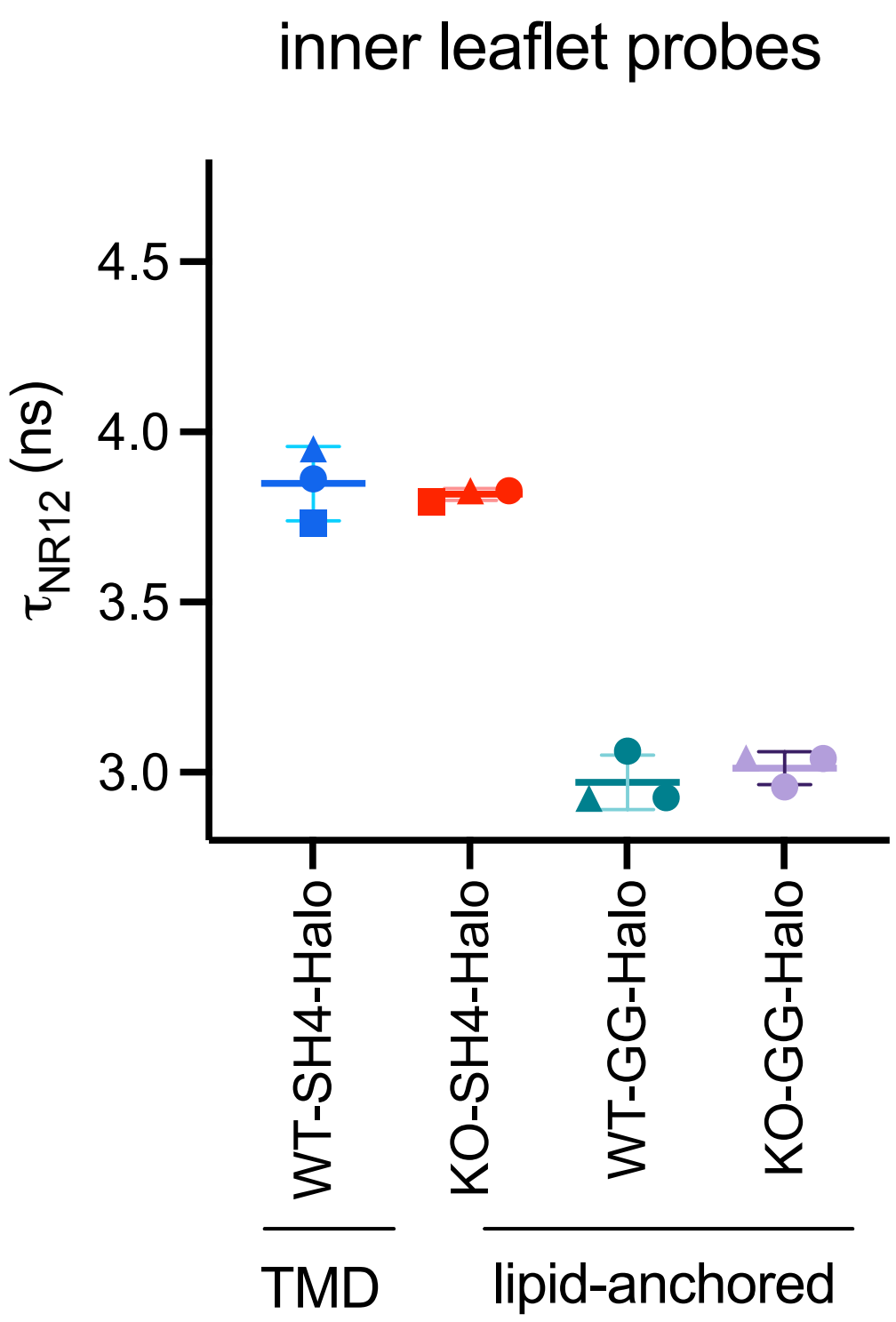

Fig. S2. Lipid order in the inner leaflet does not change notably. HaloTag-Nile Red was used to measure local lipid environment of three reporter proteins: one ordered domain (SH4) and one disordered domain (GG) preferring. Lifetime of Nile Red is shown for three peptides. Each point is a replicate.

Journal of Cell Science • Supplementary information

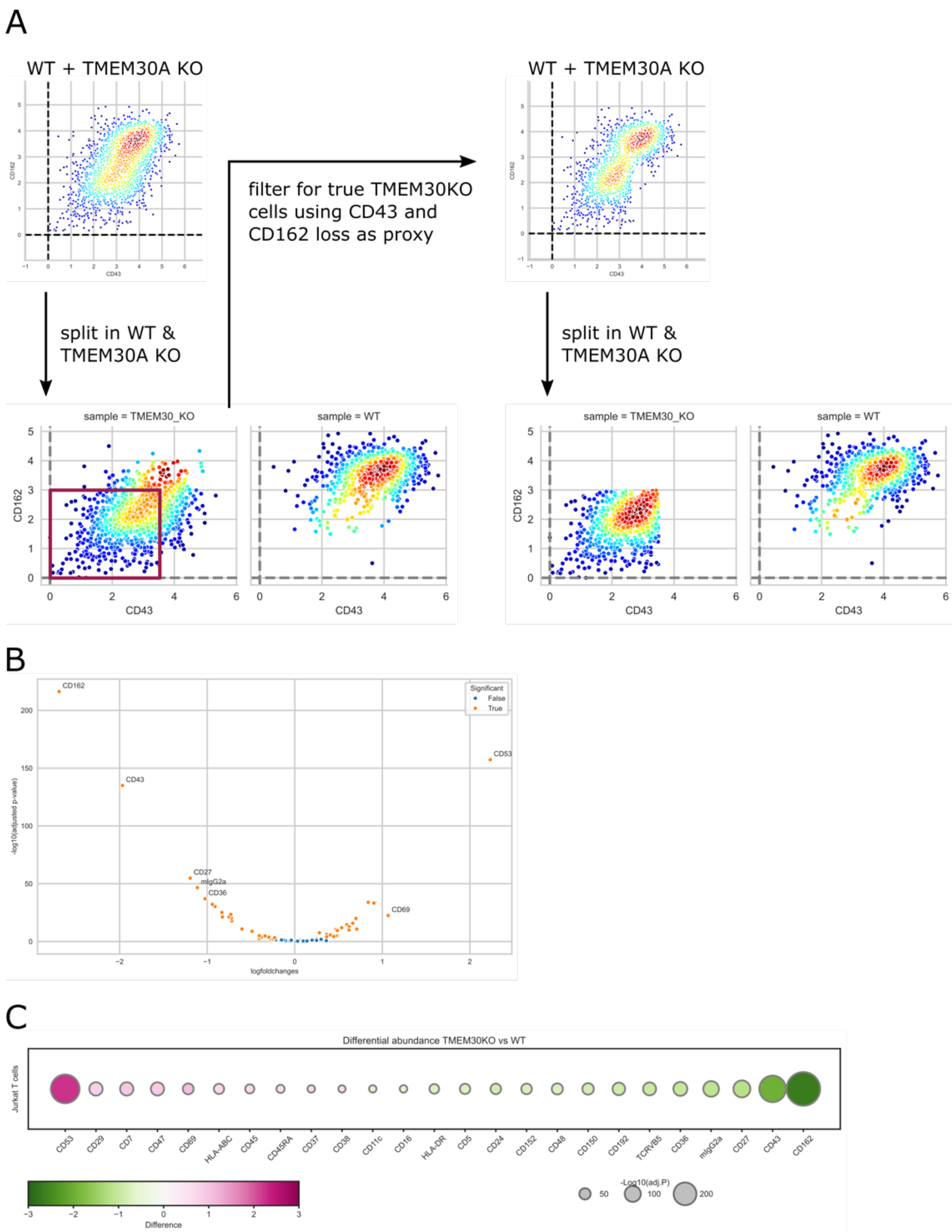

Fig. S3. Surface protein abundance after filtering for true TMEM30A KO cells. A) TMEM30A KO cells are a cell pool and not all cells have the KO as indicated by cells expressing CD43 and CD162. TMEM30A KO cells are filtered for cells with a shifted clr value of CD162<3 and CD43>3.5. After filtering two distinct cell populations are visible. B) Volcano plot for the abundance of proteins differentially regulated in WT vs. true KO Jurkat cells. Orange dots indicate significantly up- or downregulated proteins (adjusted p-value < 0.01). Labels are added for proteins with a log fold change > 1. C) Proteins of differential abundance (log fold change >0.6) in WT vs. true KO Jurkat cells. Color corresponds log fold change and size of the bubbles indicates the adjusted p-value.

Journal of Cell Science • Supplementary information

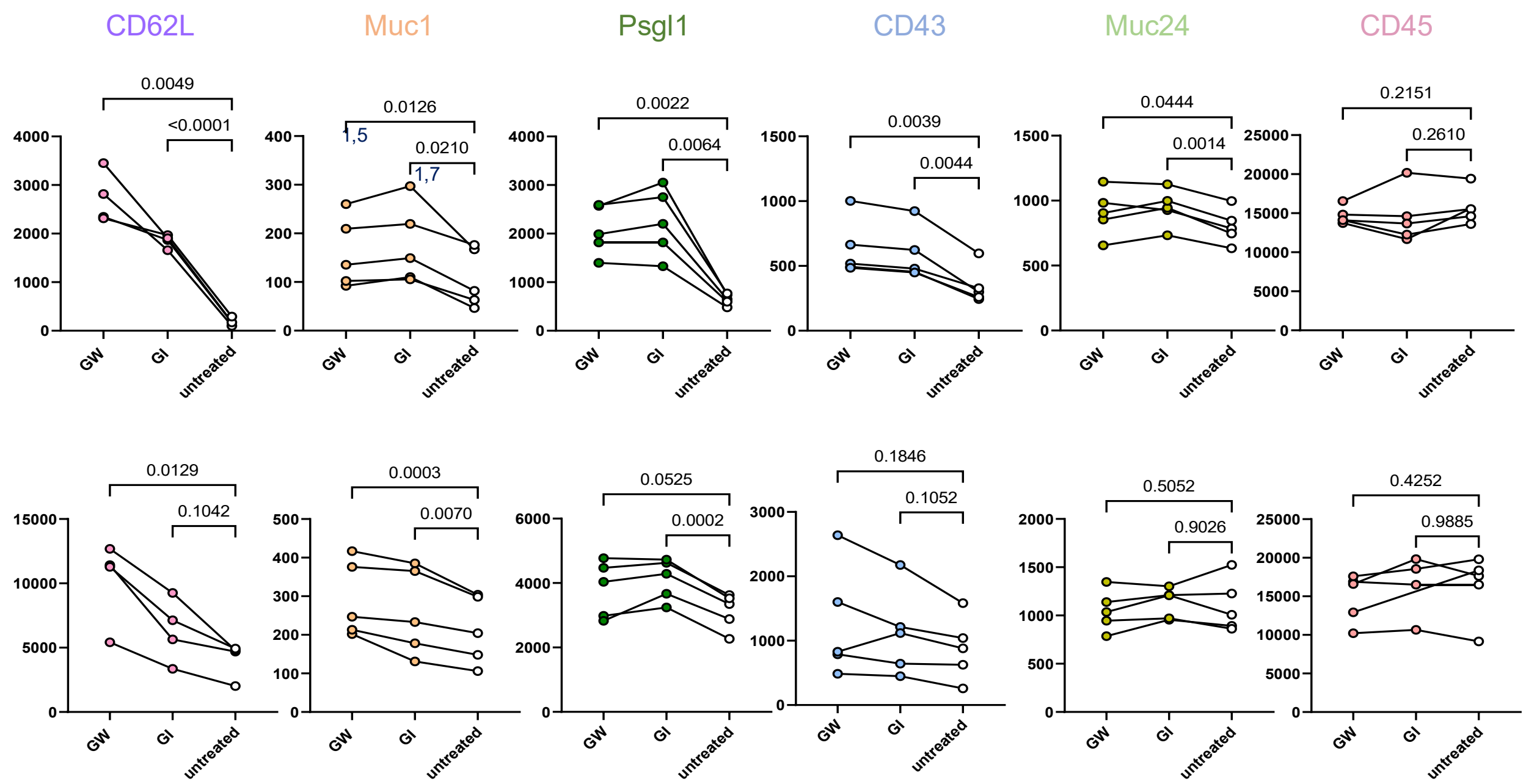

Fig. S4. The protein shedding by ADAM10 in Jurkat WT and TMEM30A-KO cells. Top panel shows the TMEM30A-KO cells and bottom panel is WT cells. ADAM10 inhibitor GI254023X (GI) and ADAM family inhibitor GW280264X (GW) reduces the shedding significantly in TMEM30A-KO cells and notably in WT cells.

Journal of Cell Science • Supplementary information
