# Supplementary material for: Cell surface remodeling caused by the loss of the flippase subunit TMEM30A in immune cells
Source: J Cell Sci. 2026 Aug 14;139(21):jcs264813. doi: 10.1242/jcs.264813 (PMC13535326; doi:10.1242/jcs.264813)
Supplement: Supplementary file 2 [file joces-139-264813_review_history.pdf]

Journal of Cell Science

Cell surface remodeling caused by the loss of flippase subunit TMEM30A in immune cells

Cenk O. Gurdap, Franziska Ragaller, Marion Mueller, Ellen Sjule, Taras Sych, Linnea Blomén, Fredrik B. Thorén, Ilya Levental, Kandice R. Levental, Quentin J. Sattentau and Erdinc Sezgin
DOI: 10.1242/jcs.264813

Editor: Francesca Bottanelli

Review timeline

Original submission:	21 February 2026
Editorial decision:	7 April 2026
First revision received:	10 June 2026
Editorial decision:	23 June 2026
Second revision received:	24 June 2026
Accepted:	24 June 2026

Original submission

First decision letter

MS ID#: jcs.264813

MS TITLE: Cell surface remodeling caused by the loss of TMEM30A in immune cells

AUTHORS: Cenk O Gurdap; Franziska Ragaller; Marion Mueller; Ellen Sjule; Taras Sych; Linnea Blomen; Fredrik Thoren; Ilya Levental; Kandice Levental; Quentin Sattentau; Erdinc Sezgin
ARTICLE TYPE: Short Report

Dear Dr Sezgin,

We have now reached a decision on the above manuscript.

As you will see, the reviewers gave favourable reports but raised some minor concerns about the interpretation of the results. In addition, they are requiring some clarifications on experimental procedures.

Reviewer 1

In the manuscript by Gurdap et al., the authors investigate the consequences of TMEM30A loss in Jurkat T cells (and partly in K562 leukemia cells) and propose that disruption of phospholipid asymmetry leads to remodelling of the plasma membrane. Using a combination of lipid reporters, membrane order probes, diffusion experiments, and surface proteome mapping via molecular pixelation, the study claims that TMEM30A knockout cells show increased phosphatidylserine exposure, increased membrane fluidity, decreased membrane order, and extensive changes in the surface proteome, including increased tetraspanins and CD47 as well as decreased mucins. The authors further propose that PS exposure activates ADAM10-dependent mucin shedding, leading to remodelling of the glycocalyx and increased accessibility of the cell surface.

© 2026. Published by The Company of Biologists under the terms of the Creative Commons Attribution License (https://creativecommons.org/licenses/by/4.0/).

The manuscript is clearly written, well structured, and addresses an interesting biological question, namely how loss of lipid asymmetry affects the physical and molecular organization of the plasma membrane. The combination of membrane biophysics and surface proteomics is highly valuable in my view.

However, several aspects of the interpretation appear overstated relative to the data, and some methodological and conceptual points require clarification. In its current form, the manuscript would benefit from a more cautious interpretation:

Major comments

Mechanistic link between PS exposure and ADAM10 activation A central claim of the manuscript is that TMEM30A loss causes PS externalization, which in turn activates ADAM10 and leads to shedding of mucins from the cell surface. While the experiments clearly show PS exposure and reduced mucin levels in TMEM30A-KO cells, the direct activation of ADAM10 by PS is not demonstrated in this study. The conclusion relies largely on previous reports describing PS-dependent regulation of ADAM proteases.

Although the inhibitor experiments support the involvement of ADAM10 in mucin shedding, they do not establish that PS exposure itself is responsible for activating ADAM10 in this system. Inducing PS exposure independently of TMEM30A loss (for example through controlled lipid scrambling or other methods that increase outer-leaflet PS) and testing whether this is sufficient to trigger mucin shedding would provide stronger support for the proposed mechanism. Additional evidence for ADAM10 activation (e.g. cleavage assays or activity measurements) would strengthen this mechanistic link. At minimum, the conclusions regarding PS-driven ADAM10 activation should be presented more cautiously, since suggested experiments might turn out to be very complex (and the basis for another publication).

Interpretation regarding immune evasion The manuscript frequently connects the observed surface remodelling to immune evasion and therapeutic responses. However, the experiments presented here do not directly measure immune cell killing, immune synapse formation, or phagocytosis. The link between the measured membrane properties and immune evasion therefore remains speculative.

In fact, the authors themselves note that the observed decrease in membrane order would be expected to increase susceptibility to immune cell killing rather than protect against it. The subsequent explanation invoking CD47 upregulation and glycocalyx changes is interesting but remains hypothetical. These interpretations should therefore be framed more clearly as hypotheses rather than firm conclusions.

Diffusion analysis using FCS The manuscript states that diffusion curves were fitted using a three-dimensional diffusion model. Since the measurements are performed at the equatorial plane of T cells using a lipid tracer, diffusion is expected to occur predominantly in two dimensions. It would therefore be helpful if the authors clarify why a 3D diffusion model was used instead of a 2D model, and how this choice affects the extracted diffusion coefficients. However I agree, that it the comparative results might not be effected by the model chosen.

Context to other literature The discussion could benefit from placing the observed membrane remodelling into the context of previous work showing that perturbations of lipid composition and lipid asymmetry can disrupt plasma membrane organization. For example, the Schütz lab (10.1016/j.bpj.2015.11.018 Biophys. J., 2015) demonstrated that treatment of cells with oxidized phospholipids leads to phosphatidylserine (PS) exposure at the outer leaflet together with dissolution of plasma membrane nanoplatforms and increased lipid mobility. Although the mechanism is different from the genetic loss of TMEM30A described here. Including this work in the discussion could therefore help place the current findings into a broader biophysical context and strengthen the link between lipid asymmetry and membrane domain organization.

Minor comments

© 2026. Published by The Company of Biologists under the terms of the Creative Commons Attribution License (https://creativecommons.org/licenses/by/4.0/).

1. Some minor typos: e.g. "TMEM30A-deficieint", "this increases can account for", "anto-CD47", "670-688 m, (center 670..."

2. The methods section refers to previous publications for experimental details. While this is acceptable, a brief description of key experimental procedures would improve reproducibility.

3. The number of biological replicates in several experiments appears relatively small (often n = 3), however I assume that every replicate contains a lot of cells. It would be helpful if the authors clarify how many cells were included in the analysis.

Reviewer 2

The manuscript by Gurdap et al. characterizes the impact of TMEM30A loss in immune cells. The authors find that in addition to the expected PS scrambling phenotype, TMEM30A loss results in decreased plasma membrane order and glycocalyx remodeling. These results are important, as they suggest functions of TMEM30A/lipid asymmetry besides the canonical role of maintaining plasma membrane structure/lipid distribution. The glycocalyx remodeling assayed by molecular pixelation is fully in line with our recent whole-cell proteome data (Barahtjan et al. BioRxiv 2025) where we find significant changes to cell-cell adhesion complexes and overall glycosylation processes. Having orthogonal methodologies in very different cell types point to the same pathways is always a welcome sign of the generality of the findings. Overall, I like the paper and recommend its acceptance after a few minor changes in data presentation/formulations.

Results:

- In figure 1 images it appears as if there is significant cell-to cell variability of LactC2-recruitment. I recommend to also report this in the quantification, Joachim Goedhard's superplots may be one option for visualization. I'd also prefer a non-normalized quantification - currently each experiment is normalized independently, which completely masks variability in the WT conditions

- The authors find that the order of the inner membrane leaflet does not change much, implying that the SLs remain on the outer leaflet. Thus, the phospholipid number on the outer leaflet is increasing relative to the inner leaflet and missing phospholipids on the inner leaflet would be replaced by cholesterol. To which extend would PS/PE have to equilibrate between the bilayers to explain the observed changes in membrane biophysical properties? Given the expertise of the authors and their significant body of work on membrane transbilayer organization I believe that they can estimate a number and add a few lines of discussion. I mainly make this statement as I intuitively feel that the arrival of ~10-15% polyunsaturated phospholipids on the OL will have a greater effect than the arrival of a similar amount of cholesterol on the inner leaflet, if SLs are unaffected. Thus, I suspect that a "tighter regulation" of the IL as stated by the authors (Lines 166ff) is even necessary from a pure membrane order point of view (there may be a replacement of PS by PA, but we don't see that in our lipidomics data of a TMEM30A KD in HCT cells that shows a similar PS externalization phenotype). I may be wrong - this is the authors area of expertise, but without further evidence I would not be speculating about potential feedback mechanisms & rather focus on the notion that not all PS asymmetry disruptions are created equally.

For what it is worth, I think that outer leaflet lipid composition is probably more tightly regulated than the inner leaflet - the number of lipid storage diseases that involve specific OL lipids is one interesting datapoint in this discussion.

- Line 195ff: How sure are we that glycolipids are not equally involved in cell-cell interactions? They are extremely predictive of cell identity (see Evelyn Rampler's JACS paper)

Discussion:

- I would phrase a couple of conclusions not as a logical deduction but as hypotheses, e.g., with regard to B-Cell Receptor mobility. The authors only discuss "on" rates - receptor encounters, nanocluster formation, but a stark change in membrane properties will affect all rates and the outcome on signal transduction will likely depend on many factors. Alternatively, the authors could

© 2026. Published by The Company of Biologists under the terms of the Creative Commons Attribution License (https://creativecommons.org/licenses/by/4.0/).

simply do the experiment & look at receptor activation & downstream signaling. I don't deem this necessary for this paper, but if the authors want to keep the statement "In practical terms, this means TMEM30A-deficient B cells are intrinsically more prone to initiate and sustain signaling due to diffusion-controlled interactions in the plasma membrane" I think it has to be shown in the same system, not just by correlation with previous measurements under different conditions. There is just too much complexity and variability in cellular signaling cascades.

André Nadler

First revision

Author response to reviewers' comments

We sincerely thank the reviewers and the editor for their thorough evaluation of our manuscript and for their constructive feedback. We believe that the revisions have strengthened the manuscript, reduced unwarranted speculation, and made it more accessible to a broader audience. Below, we provide detailed responses to each comment along with the corresponding changes made in the manuscript.

Reviewer #1:

The manuscript is clearly written, well structured, and addresses an interesting biological question, namely how loss of lipid asymmetry affects the physical and molecular organization of the plasma membrane. The combination of membrane biophysics and surface proteomics is highly valuable in my view. However, several aspects of the interpretation appear overstated relative to the data, and some methodological and conceptual points require clarification. In its current form, the manuscript would benefit from a more cautious interpretation:

We thank Reviewer 1 for overall positive comments. We addressed their concerns below.

Mechanistic link between PS exposure and ADAM10 activation

A central claim of the manuscript is that TMEM30A loss causes PS externalization, which in turn activates ADAM10 and leads to shedding of mucins from the cell surface. While the experiments clearly show PS exposure and reduced mucin levels in TMEM30A-KO cells, the direct activation of ADAM10 by PS is not demonstrated in this study. The conclusion relies largely on previous reports describing PS-dependent regulation of ADAM proteases. Although the inhibitor experiments support the involvement of ADAM10 in mucin shedding, they do not establish that PS exposure itself is responsible for activating ADAM10 in this system. Inducing PS exposure independently of TMEM30A loss (for example through controlled lipid scrambling or other methods that increase outer-leaflet PS) and testing whether this is sufficient to trigger mucin shedding would provide stronger support for the proposed mechanism. Additional evidence for ADAM10 activation (e.g. cleavage assays or activity measurements) would strengthen this mechanistic link. At minimum, the conclusions regarding PS-driven ADAM10 activation should be presented more cautiously, since suggested experiments might turn out to be very complex (and the basis for another publication).

Although the effect of PS on ADAM family activity in different biological scenarios (such as apoptosis) is well-supported in the literature, we have tuned down our claims as such experiments with well-controlled PS scrambling is complex. We agree that any means to increase PS externalization brings different complexity and unambiguously showing that PS directly causes ADAM activation is not straightforward. While we are exploring ways to show this, we will at present follow the reviewer's advice and present our data more cautiously in this manuscript, avoiding overstatement. We have now made clear that "PS externalization directly or indirectly leads to ADAM10 activation" (revised manuscript, lines 78-79, 111-113, 208-212, 283-284, 345-346) instead of claiming that PS directly causes ADAM10 activation. We also cite key references showing PS exposure strongly correlates with ADAM10 activity in different scenarios such as during apoptosis and we

© 2026. Published by The Company of Biologists under the terms of the Creative Commons Attribution License (https://creativecommons.org/licenses/by/4.0/).

state that direct PS-dependent ADAM10 activation remains to be definitively demonstrated.

Interpretation regarding immune evasion

The manuscript frequently connects the observed surface remodelling to immune evasion and therapeutic responses. However, the experiments presented here do not directly measure immune cell killing, immune synapse formation, or phagocytosis. The link between the measured membrane properties and immune evasion therefore remains speculative. In fact, the authors themselves note that the observed decrease in membrane order would be expected to increase susceptibility to immune cell killing rather than protect against it. The subsequent explanation invoking CD47 upregulation and glycocalyx changes is interesting but remains hypothetical. These interpretations should therefore be framed more clearly as hypotheses rather than firm conclusions.

We agree with the reviewer, therefore, we tuned down the conclusions and presented these as speculations in both “Results and Discussion” and in “Conclusion” sections.

Diffusion analysis using FCS

The manuscript states that diffusion curves were fitted using a three-dimensional diffusion model. Since the measurements are performed at the equatorial plane of T cells using a lipid tracer, diffusion is expected to occur predominantly in two dimensions. It would therefore be helpful if the authors clarify why a 3D diffusion model was used instead of a 2D model, and how this choice affects the extracted diffusion coefficients. However I agree, that it the comparative results might not be effected by the model chosen.

We indeed used a 2D fitting model, we corrected this mistake.

Context to other literature

The discussion could benefit from placing the observed membrane remodelling into the context of previous work showing that perturbations of lipid composition and lipid asymmetry can disrupt plasma membrane organization. For example, the Schütz lab (10.1016/j.bpj.2015.11.018 Biophys. J., 2015) demonstrated that treatment of cells with oxidized phospholipids leads to phosphatidylserine (PS) exposure at the outer leaflet together with dissolution of plasma membrane nanoplatforms and increased lipid mobility. Although the mechanism is different from the genetic loss of TMEM30A described here. Including this work in the discussion could therefore help place the current findings into a broader biophysical context and strengthen the link between lipid asymmetry and membrane domain organization.

We appreciate for flagging this up. We now discussed this and other relevant literature in the revised version in the conclusion section (lines 425-435).

Minor comments

1. Some minor typos: e.g. "TMEM30A-deficieint", "this increases can account for", "anto-CD47", "670-688 m, (center 670..."

We apologize for the typos; we corrected them in the revised manuscript.

2. The methods section refers to previous publications for experimental details. While this is acceptable, a brief description of key experimental procedures would improve reproducibility.

We agree, therefore, we added all the details to the methods.

3. The number of biological replicates in several experiments appears relatively small (often n = 3), however I assume that every replicate contains a lot of cells. It would be helpful if the authors clarify how many cells were included in the analysis.

It is correct, while n=3 are biological replicates, each replicate has many cells analyzed (100s in microscopy and 1000s for flow cytometry). We have now specified this in the revised manuscript.

© 2026. Published by The Company of Biologists under the terms of the Creative Commons Attribution License (https://creativecommons.org/licenses/by/4.0/).

Reviewer #2:

The manuscript by Gurdap et al. characterizes the impact of TMEM30A loss in immune cells. The authors find that in addition to the expected PS scrambling phenotype, TMEM30A loss results in decreased plasma membrane order and glycocalyx remodeling. These results are important, as they suggest functions of TMEM30A/lipid asymmetry besides the canonical role of maintaining plasma membrane structure/lipid distribution. The glycocalyx remodeling assayed by molecular pixelation is fully in line with our recent whole-cell proteome data (Barahtjan et al. BioRxiv 2025) where we find significant changes to cell-cell adhesion complexes and overall glycosylation processes. Having orthogonal methodologies in very different cell types point to the same pathways is always a welcome sign of the generality of the findings. Overall, I like the paper and recommend its acceptance after a few minor changes in data presentation/formulations.

We are grateful for the reviewer for positive comments and his effort. We are also very glad to see that our findings are in line with the reviewer's lab's very relevant manuscript. We cited and discussed this work now in Discussion section.

- In figure 1 images it appears as if there is significant cell-to cell variability of LactC2- recruitment. I recommend to also report this in the quantification, Joachim Goedhard's superplots may be one option for visualization. I'd also prefer a non-normalized quantification - currently each experiment is normalized independently, which completely masks variability in the WT conditions

Panel B is from flow cytometry experiments which includes around 10000 cells per replicate, therefore showing all non-normalized data is not really possible, hence we show the medians. However we now present all replicates separately with absolute values (as violin plots) for both LactC2 and Pro12A as a new Supplementary Figure 1.

- The authors find that the order of the inner membrane leaflet does not change much, implying that the SLs remain on the outer leaflet. Thus, the phospholipid number on the outer leaflet is increasing relative to the inner leaflet and missing phospholipids on the inner leaflet would be replaced by cholesterol. To which extend would PS/PE have to equilibrate between the bilayers to explain the observed changes in membrane biophysical properties? Given the expertise of the authors and their significant body of work on membrane transbilayer organization I believe that they can estimate a number and add a few lines of discussion. I mainly make this statement as I intuitively feel that the arrival of ~10-15% polyunsaturated phospholipids on the OL will have a greater effect than the arrival of a similar amount of cholesterol on the inner leaflet, if SLs are unaffected. Thus, I suspect that a "tighter regulation" of the IL as stated by the authors (Lines 166ff) is even necessary from a pure membrane order point of view (there may be a replacement of PS by PA, but we don't see that in our lipidomics data of a TMEM30A KD in HCT cells that shows a similar PS externalization phenotype). I may be wrong - this is the authors area of expertise, but without further evidence I would not be speculating about potential feedback mechanisms & rather focus on the notion that not all PS asymmetry disruptions are created equally.

We agree with the reviewer, hence we removed the: "tight regulation of inner leaflet compared to outer leaflet" speculation and focused our discussion around the heterogeneity of PS externalization mechanisms and their potential outcomes.

For the lipid numbers, it is not trivial to speculate on the number balance in this situation, therefore we have refrained from speculating on this matter.

For what it is worth, I think that outer leaflet lipid composition is probably more tightly regulated than the inner leaflet - the number of lipid storage diseases that involve specific OL lipids is one interesting datapoint in this discussion.

- Line 195ff: How sure are we that glycolipids are not equally involved in cell-cell interactions? They are extremely predictive of cell identity (see Evelyn Rampler's JACS paper).

We changed the statement to "a major determinant" rather than "the central determinant".

© 2026. Published by The Company of Biologists under the terms of the Creative Commons Attribution License (https://creativecommons.org/licenses/by/4.0/).

Discussion:

- I would phrase a couple of conclusions not as a logical deduction but as hypotheses, e.g., with regard to B-Cell Receptor mobility. The authors only discuss "on" rates - receptor encounters, nanocluster formation, but a stark change in membrane properties will affect all rates and the outcome on signal transduction will likely depend on many factors. Alternatively, the authors could simply do the experiment & look at receptor activation & downstream signaling. I don't deem this necessary for this paper, but if the authors want to keep the statement "In practical terms, this means TMEM30A-deficient B cells are intrinsically more prone to initiate and sustain signaling due to diffusion-controlled interactions in the plasma membrane" I think it has to be shown in the same system, not just by correlation with previous measurements under different conditions. There is just too much complexity and variability in cellular signaling cascades.

We agree and have tuned-down such statements as both reviewers suggested. We now clearly state that these are speculations or hypothesis and will require further work to be tested (conclusions section lines 375-381 and 421-424).

Second decision letter

MS ID#: jcs.264813R1

MS TITLE: Cell surface remodeling caused by the loss of flippase subunit TMEM30A in immune cells

AUTHORS: Cenk O Gurdap; Franziska Ragaller; Marion Mueller; Ellen Sjule; Taras Sych; Linnea Blomen; Fredrik Thoren; Ilya Levental; Kandice Levental; Quentin Sattentau; Erdinc Sezgin
ARTICLE TYPE: Research Article

Dear Dr Sezgin,

We have now reached a decision on the above manuscript.

As you will see from the reviewers' comments, they are satisfied with the revisions made to the manuscript. Only a minor textual amendment suggested by Reviewer 2 remains to be addressed. Please revise the manuscript accordingly, after which I will be pleased to recommend it for final acceptance.

As discussed, we would like to publish your article as a Report. However, no modifications to the manuscript length are necessary from your side.

Reviewer 1

The authors have satisfactorily addressed all comments raised during the initial review. The revisions have improved the clarity of the manuscript, reduced instances of overinterpretation, and strengthened the discussion and presentation of the results. I have no further concerns and believe that the manuscript is now suitable for publication.

Reviewer 2

I recommend acceptance of the article in its revised form, the authors addressed all my points

Minor comments:

© 2026. Published by The Company of Biologists under the terms of the Creative Commons Attribution License (https://creativecommons.org/licenses/by/4.0/).

Technically, we do do not actually show "downregulation of glycosylation" biochemically in the Barahtjan et al preprint, but a downregulation of glycosylation pathways in transcriptomic/proteomic data. Maybe adapt the sentence by one word?

André Nadler

Second revision

Author response to reviewers' comments

Reviewer 2:

Technically, we do do not actually show "downregulation of glycosylation" biochemically in the Barahtjan et al preprint, but a downregulation of glycosylation pathways in transcriptomic/proteomic data. Maybe adapt the sentence by one word?

We changed the text to *"Interestingly, downregulation of overall glycosylation pathways in transcriptome and proteome was also observed in adherent cells with flippase knock- down adherent cells (Barahtjan et al., 2025)."*

Third decision letter

MS ID#: jcs.264813R2

MS Title: Cell surface remodeling caused by the loss of flippase subunit TMEM30A in immune cells

Authors: Cenk O Gurdap; Franziska Ragaller; Marion Mueller; Ellen Sjule; Taras Sych; Linnea Blomen; Fredrik Thoren; Ilya Levental; Kandice Levental; Quentin Sattentau; Erdinc Sezgin
Article Type: Short Report

Dear Dr Sezgin,

I am happy to tell you that your manuscript has been accepted for publication in Journal of Cell Science, pending standard publication integrity checks.

© 2026. Published by The Company of Biologists under the terms of the Creative Commons Attribution License (https://creativecommons.org/licenses/by/4.0/).
